# Altered hypothalamic DNA methylation and stress-induced hyperactivity following early life stress

**DOI:** 10.1186/s13072-021-00405-8

**Published:** 2021-06-30

**Authors:** Eamon Fitzgerald, Matthew C. Sinton, Sara Wernig-Zorc, Nicholas M. Morton, Megan C. Holmes, James P. Boardman, Amanda J. Drake

**Affiliations:** 1 University/British Heart Foundation Centre for Cardiovascular Science, University of Edinburgh, The Queens Medical Research Institute, 47 Little France Crescent, Edinburgh, EH16 4TJ UK; 2 Department of Biochemistry III, University of Regensburg, 93040 Regensburg, Germany; 3 MRC Centre for Reproductive Health, University of Edinburgh, The Queens Medical Research Institute, 47 Little France Crescent, Edinburgh, EH16 4TJ UK; 4 Centre for Clinical Brain Sciences, University of Edinburgh, Chancellor’s Building, 49 Little France Crescent, Edinburgh, EH16 4SB UK; 5 The Douglas Research Center, 6875 Boulevard LaSalle, Montréal, QC H4H 1R3 Canada

**Keywords:** Early life stress, DNA methylation, Behaviour, Hypothalamus, Brain development, Preterm birth

## Abstract

Exposure to early life stress (ELS) during childhood or prenatally increases the risk of future psychiatric disorders. The effect of stress exposure during the neonatal period is less well understood. In preterm infants, exposure to invasive procedures is associated with altered brain development and future stress responses suggesting that the neonatal period could be a key time for the programming of mental health. Previous studies suggest that ELS affects the hypothalamic epigenome, making it a good candidate to mediate these effects. In this study, we used a mouse model of early life stress (modified maternal separation; MMS). We hypothesised MMS would affect the hypothalamic transcriptome and DNA methylome, and impact on adult behaviour. MMS involved repeated stimulation of pups for 1.5 h/day, whilst separated from their mother, from postnatal day (P) 4–6. 3’mRNA sequencing and DNA methylation immunoprecipitation (meDIP) sequencing were performed on hypothalamic tissue at P6. Behaviour was assessed with the elevated plus, open field mazes and in-cage monitoring at 3–4 months of age. MMS was only associated with subtle changes in gene expression, but there were widespread alterations in DNA methylation. Notably, differentially methylated regions were enriched for synapse-associated loci. MMS resulted in hyperactivity in the elevated plus and open field mazes, but in-cage monitoring revealed that this was not representative of habitual hyperactivity. ELS has marked effects on DNA methylation in the hypothalamus in early life and results in stress-specific hyperactivity in young adulthood. These results have implications for the understanding of ELS-mediated effects on brain development.

**Supplementary Information:**

The online version contains supplementary material available at 10.1186/s13072-021-00405-8.

## Introduction

Experiencing early life stress (ELS) has been associated with an increased risk of psychiatric disorders including depression and anxiety in adulthood [1–3]. Individuals with a psychiatric disorder who were exposed to ELS are also less likely to respond well to treatment compared to those who were not [4, 5], perhaps indicating distinct mechanisms by which ELS can program future outcomes. A growing number of studies have reported ELS during childhood increases the risk for future psychiatric disorders [6, 7] and there are well-described long-term adverse consequences of prenatal exposure to maternal stress on mental health in offspring [8–10]. However, less is known about the long-term effects of ELS experienced specifically during the neonatal period. Most studies addressing stressors during the perinatal period have been undertaken in babies born preterm (birth at less than 37 weeks of gestation), with previous studies suggesting that stressful events such as exposure to painful procedures can affect brain development [11] and programme future stress responses [12].

One potential mechanism by which ELS may act is through programming of the HPA axis [13] and studies of ELS in animal models have described altered stress reactivity, neuronal activation and differences in DNA methylation within the hypothalamus [14–17], which is a key mediator of the ELS response [17, 18]. Such studies have led to the proposal that changes in DNA methylation may at least partially mediate the effects of ELS, through influencing transcriptional events [19]. Moreover, several stress-related stimuli including glucocorticoids [20] and neuronal activity can affect DNA methylation [21]. In humans, changes in DNA methylation have been seen in post-mortem hippocampal tissue from individuals exposed to ELS [22] and peripheral DNA methylation around the *FKBP5* gene has been proposed to mediate the response to ELS in humans [23].

To better understand the influence of ELS on neurodevelopment and to dissect the underlying mechanisms, animal models have been used [24]. Various ELS models, including maternal separation and altered maternal care have been associated with an array of different phenotypes, including effects on the hypothalamic–pituitary–adrenal (HPA) axis and vulnerability to adult stress [24]. Maternal separation is the most common model of ELS which involves pup separation from the mother for ≥ 3 h/day for ≥ 10 consecutive days however considering the mother is the sole source of nutrition during this time, nutritional deficits may confound any findings in this model as hypoglycemia is independently associated with impaired neurodevelopment in humans [25]. To avoid this we used a modified maternal separation paradigm (MMS) to model ELS, involving short periods (1.5 h/day from postnatal day (P)4–P6 of brief maternal separation in combination with manual manipulation during the period of separation [26].

We hypothesised that MMS would result in perturbations of the hypothalamic transcriptome and DNA methylome and in altered stress-induced behaviour in adulthood. We tested this using a combination of candidate gene expression analysis (for genes involved in glucocorticoid signalling and DNA methylation), 3’mRNA sequencing and DNA methylation immunoprecipitation (meDIP) sequencing in the hypothalamus at P6 (immediately following MMS). We then performed behavioural assessment using the elevated plus maze (EPM), open field (OF) and in-cage behavioural analysis of habitual activity at 3–4 months of age in adult male mice. We found that MMS associates with profound changes in hypothalamic DNA methylation in the neonatal period and with a stress-specific hyperactive phenotype in adulthood.

## Methods and materials

### Animals

Experiments were carried out in accordance with University of Edinburgh guidelines and the UK Home Office Animals (Scientific Procedures) Act 1986. Adult C57/Bl6 mice (Harlan, UK) had ad libitum access to chow (Special Diets Services, Essex, UK) and water (lights on 07:00–19:00, temperature 22 °C). For mating, 2 females and 1 male were kept per cage. Dams were checked daily for new litters with P0 designated as the day of birth. Mice were weaned at P21 with littermates housed together. Neonatal experiments consisted of *n* = 10/group (10 independent litters), unless otherwise stated. Adult experiments were performed on *n* = 11/group (11 independent litters) for biochemistry, elevated plus maze (EPM), open field (OF) and tail suspension and *n* = 7 for in-cage behavioural analysis.

### Modified maternal separation (MMS) and behavioural testing

At P3, the litters were reduced leaving 4 male pups only. Within a litter, two males were randomly assigned to the control group and two to MMS. As previously described [26], MMS was performed between 1330 and 1500 daily from P4–P6 in a different room to their resident holding room. MMS pups were placed on a heating pad adjacent to the home-cage and for 1.5 h, pups were gently moved to the supine position whenever they returned to a prone position. Pups were then returned to the home-cage. Pups were weighed daily; weights were normalised to P4 weight.

Immediately after MMS on P6, one cohort was killed by decapitation. Trunk blood was collected, and blood glucose measured immediately using the AccuCheck Performa Glucometer (Roche, UK). Whole blood was collected in EDTA-coated tubes (Sarstedt, Germany) and plasma isolated and stored at − 80 °C. Whole brains were extracted and the hypothalamus dissected using the optic chiasm and the mamillary body recess as landmarks. Tissue was snap-frozen on dry ice and stored at − 80 °C.

A second cohort of mice was weaned at P21, with control and MMS littermates housed together. Behavioural testing was performed at P90–P100. The EPM was used as described [27]. Mice were placed in the centre zone facing the open arm and left to explore the maze for 5 min. The OF test was carried out as described [28], 24 h after the EPM. Mice were placed in the centre of the OF and allowed to explore for 5 min. Recording and analyses were performed automatically using the AnyMaze software (AnyMaze, Dublin). One hour after the OF, tail suspension testing was performed as described [29] for 6 min with the tester hidden. Recordings were analysed for time immobile with the investigator blinded to group.

At P120–P130, in-cage behaviour was analysed using the TSE-systems PhenoMaster (TSE-Systems, Germany). Mice were single-housed for 4 days for acclimatisation and then introduced into a fresh home-cage for testing. The mice were allowed to adjust to the novel environment for 24 h before measurements were taken. Activity measurements were automatically recorded through sensors over two consecutive 24-h periods, using detectors along the x- and y-planes. Data were averaged across 2 consecutive 24-h periods. Animals were killed by cervical dislocation. The brain was removed, leaving the pituitary gland in the skull for collection. The brain was then divided along the midline and the cortex was resected before hippocampal removal. The body cavity was then opened and the adrenal gland isolated from any surrounding fat. Tissue was frozen on dry ice and stored at − 80 °C.

### Corticosterone ELISA

Blood samples were collected by tail venesection at 7 am and 7 pm the day after the tail suspension test. Plasma corticosterone was analysed by ELISA (Enzo Life Sciences, Exeter).

### DNA/RNA extractions

DNA/RNA were extracted from the same sample using the Qiagen All Prep DNA/RNA Mini Kit (Qiagen, Manchester).

### Reverse transcription and quantitative PCR

1 µg of RNA was DNase treated with RQ1 RNase-free DNase (Promega, Hampshire). Reverse transcription was performed with the Applied Biosystems RT kit (Thermo Fisher Scientific, UK) in a G-Storm Thermocycler (Akribis Scientific Limited, Cheshire). qPCR primers were designed using the UPL assay design centre and cDNA samples analysed on a Roche LightCycler 480, normalised to the expression of the housekeeping gene *TBP*. Primers are listed in Additional file 1: Table S1.

### 3’mRNA sequencing

For 3’mRNA sequencing, 6 samples were randomly selected/group. Sequencing was performed at the Wellcome Trust Clinical Research Facility (University of Edinburgh). Library preparation was done using the QuantSeq 3’mRNA-Seq Library Prep Kit (Lexogen, Austria) and templates prepared using the Ion PI Hi-Q OT2 200 kit (Thermo Fisher Scientific, UK). Sequencing was performed using the Ion PI Chip Kit v3 (Thermo Fisher Scientific, UK), 12 samples/chip with an average of 7,822,219 reads/sample generated. This depth of sequencing has previously been shown sufficient for differential expression analysis [26, 30, 31]. The Ion Hi-Q Sequencing 200 Kit (Thermo Fisher Scientific, UK) and the Ion Proton platform were used for analysis. For data analysis, raw pH DAT files were converted to flow signals and aligned to the mm10 reference genome in an automated workflow (Torrent Suite v5.2.0). Analysis was performed using Galaxy [32] and Degust software. Gene Ontology (GO) analysis was carried as described [33, 34], using genes with a log fold change (logFC) > 1.5 [35, 36]. Transcription factor enrichment analysis was performed using oPOSSUM-3 software, with a Fisher score < 7 and a Z-score < 10 taken as significant enrichment [37]. Data are available through the Gene Expression Omnibus (GSE147375).

### DNA methylation immunoprecipitation and sequencing

For DNA methylation immunoprecipitation (meDIP) sequencing, three pooled samples/group were used. These 3 pooled samples were generated by randomly allocating the 10 independent samples/group into 3 groups, before sequencing with the Ion Proton platform [38]. This was done to increase biological diversity across sequenced samples. A mean read length of 133-144 bp and 24,288,817–34,030,252 reads/sample was achieved. Reads were aligned to the mm10 genome using Torrent Suite v5.2.0. Aligned reads were sorted using SAMtools, before calling peaks using MACS2 (v2.1.1) -f BAM –broad –broad-cutoff 0.05 -B -g hs, over corresponding inputs [39]. To detect differentially methylated regions (DMRs), we used Diffbind with DESeq2 and edgeR [40]. DMRs were assigned to genes and other genomic features using the HOMER (v4.8) annotatePeaks tool [41]. Data were normalised to a pooled input for each group and subtracted from an IgG control. Sequencing data are available through the Gene Expression Omnibus (GSE146892).

### Statistical analysis

Statistical analyses were performed using IBM SPSS software version 24. Independent t-tests were used for comparisons between control and MMS at a single timepoint. Repeated measures ANOVA was used for the comparisons between control and MMS at multiple time-points (i.e. AM and PM). For all experiments a p-value or FDR of 0.05 was taken as statistically significant.

## Results

### MMS is not associated with early alterations in weight, glucose or plasma corticosterone

**Fig. 1:**
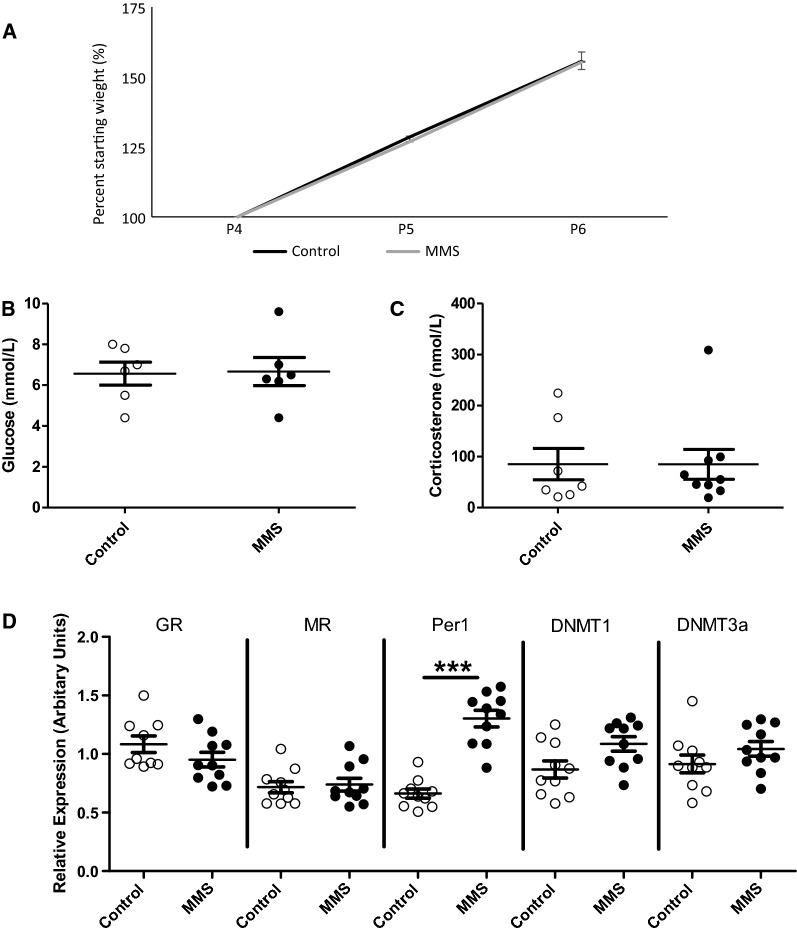
MMS is not associated with alterations in weight gain, blood glucose or plasma corticosterone, but is associated with changes in candidate gene expression in the hypothalamus. **A** There was no difference in weight gain between control (black solid line) and MMS (orange dashed line) groups during MMS (*p = *0.62 for area under the curve, df = 18), *n* = 10/group. Pup weight was standardised to 100% at the P4 timepoint. There were no differences between control (clear circles) and MMS (black filled circles), in (**B**) blood glucose (*p = *0.91) (*n* = 6/group, df = 10) or (**C**) plasma corticosterone concentrations (*p = *0.993, df = 14) immediately following MMS (*n* = 7 and 10/group). **D** Following a Bonferroni correction for multiple comparisons the threshold for statistical significance was set at *p* < 0.01. MMS was associated with increased mRNA expression of Per1 (*p = *0.009, df = 18) but DNMT1 (*p = *0.03, df = 19) fell marginally outside this threshold. There were no differences in the expression of GR (*p = *0.36, df = 17), MR (*p = *0.77, df = 18) or DNMT3a (*p = *0.21, df = 18), *n* = 10/group. All statistical comparisons were made by independent t-test. Error bars indicate standard error of the mean

In agreement with our previous report, there were no differences in weight gain (t-statistic = − 0.49, *p* = 0.62 for area under the curve, df = 18) (Fig. 1A) [26]. MMS also had no effect on blood glucose (t-statistic = − 0.11, *p* = 0.91, df = 10) (Fig. 1B) or plasma corticosterone concentrations (t-statistic = 0.009, *p* = 0.99, df = 14) (Fig. 1C) between groups. Removal of the apparent outlier from the MMS group in Fig. 1C does not change the statistical interpretation of the results.

### MMS associates with early changes in hypothalamic gene expression

Analysis of hypothalamic expression of genes involved in glucocorticoid signalling and DNA methylation revealed increased expression of the glucocorticoid responsive gene *Period 1* (*Per1*) (t-statistic = − 7.9, *p* = 0.009, df = 18), which is associated with circadian rhythms, and there was a point difference in *DNA methyltransferase 1* (*DNMT1*) expression, which did not pass the corrected p-value threshold (t-statistic = − 2.28, *p* = 0.03, df = 19) (Fig. 1D). There were no changes in the expression of the *glucocorticoid receptor* (*GR*) (t-statistic = 0.942, *p* = 0.36, df = 17), *mineralocorticoid receptor* (*MR*) (t-statistic = − 0.3, *p = *0.77, df = 18) or *DNA methyltransferase 3a* (*DNMT3a*) (t-statistic = − 1.3, *p* = 0.21, df = 18).

**Fig. 2:**
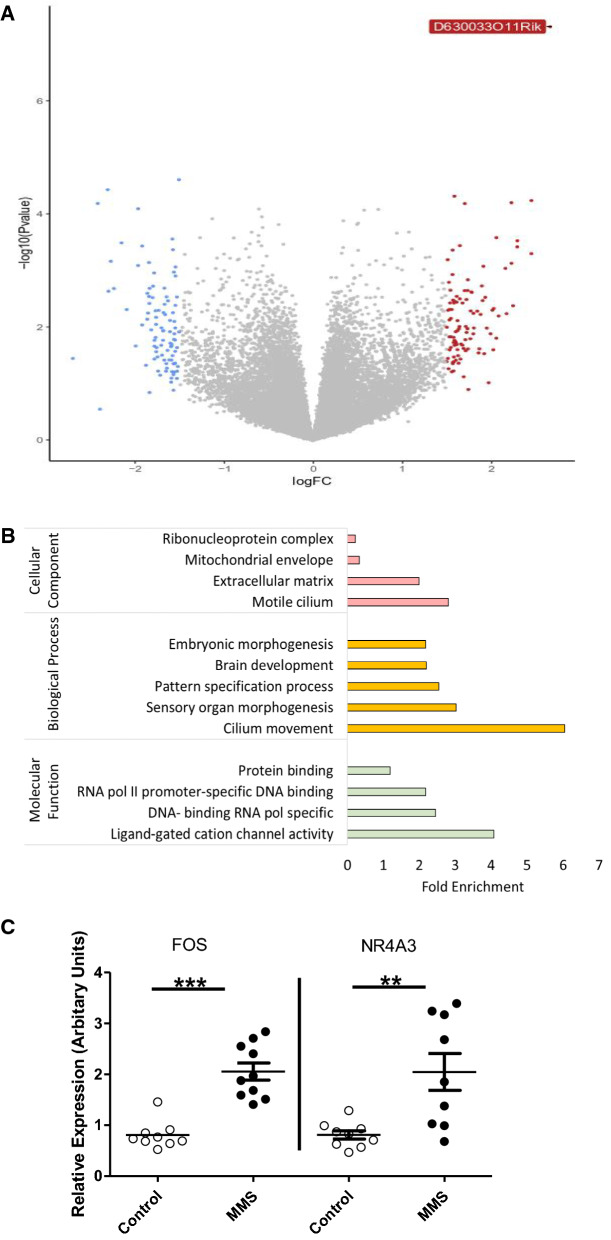
3’ mRNA sequencing of the hypothalamic transcriptome reveals only subtle changes associated with MMS. 6 samples were sequenced per group. **A** Volcano plot showing differential gene expression between groups, genes with a logFC greater than 1.5 are coloured blue (decrease) or red (increase). Only 1 gene (D630033O11Rik) had an FDR > 0.05. **B** Gene Ontology analysis of genes with a logFC > 1.5 (i.e. those coloured blue or red in **A**) for cellular component (pink), biological process (yellow) and molecular function (green). **C** Gene expression patterns, from control (clear circles) and MMS (black filled circles), identified from 3’ mRNA sequencing and Gene Ontology were validated using qPCR. FOS was enriched in the Gene Ontology term “Ligand-gated cation channel activity” and NR4A3 was identified from the enriched term “DNA-binding transcription activator activity, RNA polymerase II-specific”, in the biological process category. Both FOS (*p* > 0.001, df = 17) and NR4A3 (*p = *0.004, df = 16) were significantly increased in expression following MMS

Next we used 3’ mRNA sequencing to evaluate the whole hypothalamic transcriptome. This method has been shown to yield comparable differential expression results to that of traditional RNA sequencing methods, but with a greatly reduced sequencing depth [30, 31]. Analysis of 3’mRNA sequencing data identified only one gene (*D630033O11Rik*, labelled in Fig. 2A) as differentially expressed (FDR < 0.05). Multi-dimension scaling (MDS) showed poor clustering of groups (Additional file 2: Fig. S1A). GO analysis of genes with logFC > 1.5 [35, 36] (Fig. 2A: blue = downregulated, red = upregulated) revealed enrichment for terms associated with “DNA binding RNA polymerase specific” and “Ligand-gated cation channel activity” within the molecular function category (Fig. 2B). Genes identified in these enrichment terms with the greatest logFC were *NR4A3* and *FOS*; upregulation of both genes was validated by qPCR (Fig. 2C) (*NR4A3*; t-statistic = − 3.34, *p* = 0.004, df = 16 and *FOS*; t-statistic = − 6.37, *p* > 0.001, df = 17). Transcription factor binding enrichment analysis of genes with logFC > 1.5 showed enrichment in motifs associated with factors with neurodevelopmental functions (Additional file 2: Fig. S1B) including Nuclear Factor kappa B (NFkB) [42], Hypoxia Inducible Factor (HIF) 1α [43] and Krüppel-like factor 4 (KLF4) [44, 45]. See Additional file 1: Table S2 for a full list of genes with a logFC greater than 0.5.

### MMS associates with early alterations in hypothalamic DNA methylation

**Fig. 3:**
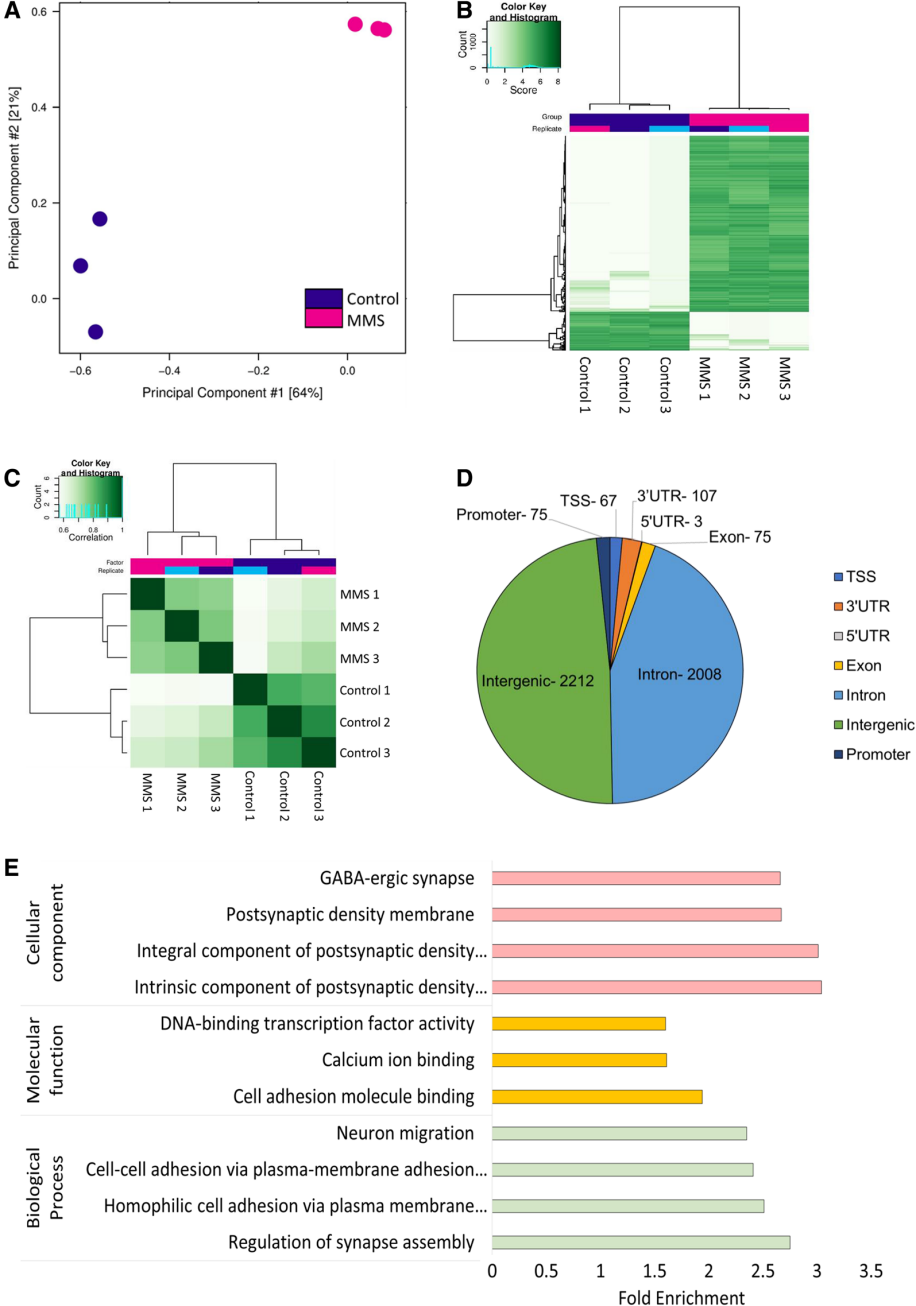
Widespread alterations in DNA methylation within the hypothalamus following MMS using meDIP sequencing. 3 samples were sequenced per group. **A** Principal component analysis of differentially methylated regions shows distinct clustering of control (blue) and MMS (pink) groups. Principal components 1 and 2 accounted for 64% and 21% of variance in the dataset, respectively. **B** Heatmap of all DMRs throughout the genome with an FDR < 0.05, which are clustered by Euclidian distance. **C** Correlation map of control and MMS samples following meDIP sequencing clustered by Euclidian distance. **D** Catalogue of the DMRs associated with protein coding regions. Numbers indicate numbers of DMRs associated with that region. (E) Gene Ontology analysis of differentially methylated sites (FDR < 0.05) associated with protein coding regions. Terms for cellular component (pink), molecular function (yellow) and biological processes (green) are shown. Notably, there is enrichment of synapse-associated terms under cellular component and biological processes

Previous research has shown ELS can affect DNA methylation [17]. As such, we postulated that DNA methylation might be affected following MMS. We investigated this using meDIP sequencing within the hypothalamus. We identified 13,000 DMRs across the genome. Principal component analysis showed a distinct effect of MMS on DNA methylation (Fig. 3A). All DMRs are represented in the heatmap depicted in Fig. 3B, clustered by Euclidian distance and within group samples were highly correlated (Fig. 3C). Next, we identified DMRs associated with protein coding regions (Fig. 3D). GO analysis revealed enrichment for terms associated with various synaptic elements (Fig. 3E). Differential methylation analysis was performed using DESeq2, but there was a substantial overlap of DMRs identified using an alternative method (edgeR) (Additional file 2: Fig. S2A). There was no correlation between DMRs associated with protein coding regions and transcript expression (Additional file 2: Fig. S2B). In particular, although DNA methylation in promoter regions is classically associated with gene expression [46], fewer than 20 promoter DMRs were identified, and these were not associated with transcript expression. See Additional file 1: Table S3 for a full list of DMRs.

### MMS is associated with hyperactivity in the elevated plus maze (EPM) and open field maze (OF) but normal habitual in-cage movement at 3–4 months

**Fig. 4:**
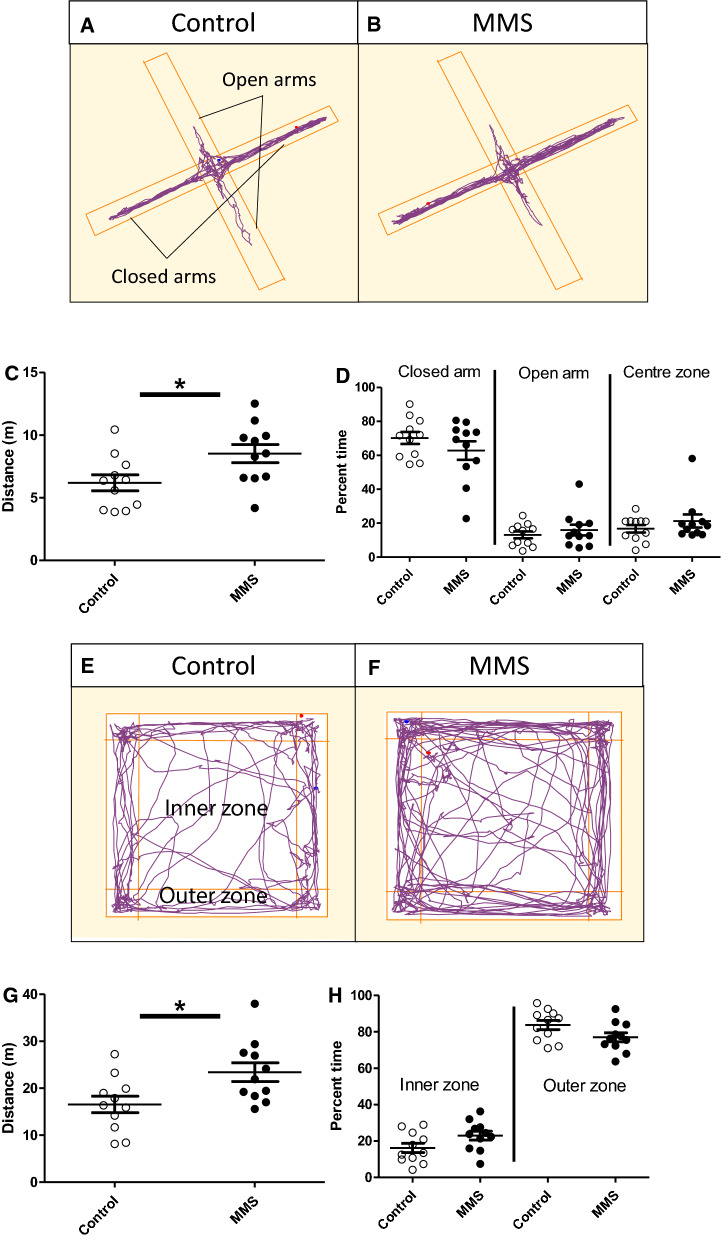
MMS is associated with hyperactivity in the elevated plus (EPM) and open field (OF) maze. (**A** and **B**) Representative track plots for control and MMS groups in the EPM, with the open and closed arms of the maze indicated in **A**. **C** The MMS group travelled further during the EPM (*n* = 11/group) (*p = *0.02, df = 20), but there were no differences in the time spent in the open (*p = *0.457, df = 20), closed (*p = *0.267, df = 20) or centre (*p = *0.32, df = 20) areas **D**. **E** and **F** Representative track plots for control and MMS groups in the OF with the inner and outer zones indicated in E. **G** The MMS group travelled further in the OF (*n* = 11/group) (*p = *0.02, df = 21) and spent similar amounts of time in the inner (*p = *0.07, df = 20) and outer zones (*p = *0.07, df = 20) (**H**). Independent t-tests were used for **C**, **D**, **G** and **H**. Error bars indicate standard error of the mean

Representative track plots for control and MMS mice in the EPM are shown in Fig. 4A, B. Mice exposed to MMS travelled further in the EPM (t-statistic = − 2.43, *p* = 0.02, df = 20) (Fig. 4C) but there were no differences in the time spent in open (t-statistic = − 0.76, *p* = 0.46, df = 20), closed (t-statistic = 1.41, *p* = 0.28, df = 20) or centre (t-statistic = – 1.02, *p* = 0.32, df = 20) areas (Fig. 4D). MMS mice also had a higher average speed (t-statistic = − 2.46, *p* = 0.02, df = 20) and total mobile time (t-statistic = − 2.49, *p* = 0.02, df = 20) (Additional file 2: Fig. 3A and B). MMS mice also travelled further in the closed arm (t-statistic = − 2.1, *p* = 0.048, df = 20), with no difference in visit duration (t-statistic = 1.94, *p* = 0.06, df = 20) (Additional file 2: Fig. 3C and D), while there were no differences in distance travelled in the open arms (t-statistic = − 0.9, *p* = 0.38, df = 20) or in visit duration (t-statistic = 0.13, *p* = 0.90, df = 20) (Additional file 2: Fig. 3E and F). Representative track plots for the control and MMS groups in the OF are shown in Fig. 4E and 4F. MMS mice travelled further (t-statistic = − 2.57, *p* = 0.02, df = 21) (Fig. 4G) but did not show altered preference for the inner (t-statistic = − 1.91, *p* = 0.07, df = 20) and outer zones (t-statistic = 1.91, *p* = 0.07, df = 20) (Fig. 4H). MMS mice also moved faster (t-statistic = − 2.57, *p* = 0.02, df = 20) and spent more time mobile (t-statistic = − 2.56, *p* = 0.02, df = 20) (Additional file 2: Fig. 4A and B). MMS mice travelled further in the OF inner (t-statistic = − 2.69, *p = *0.02, df = 20) and outer (t-statistic = − 2.15, *p* = 0.04, df = 20) zones, with a shorter length of visit to the outer zone (t-statistic = 2.11, *p* = 0.048, df = 20) (Additional file 2: Fig. 4C–F). There were no differences between groups in time immobile (t-statistic = − 1.01, *p* = 0.32, df = 20) during the tail suspension test (Additional file 2: Fig. 4G).

**Fig. 5:**
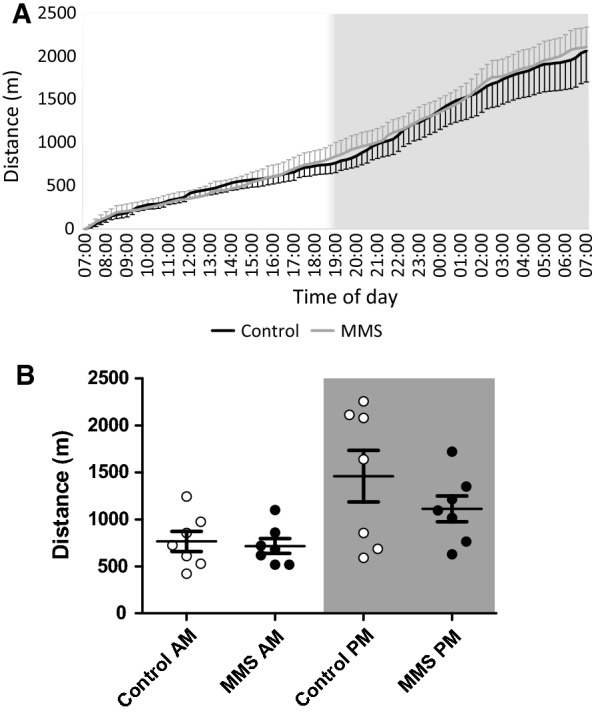
No change in habitual activity associated with MMS. In-cage behavioural monitoring using the TSE PhenoMaster system tracked movement over a 24-h period (two consecutive 24-h periods were averaged for each animal). **A** Cumulative distance travelled with respect to time of day, the light aspect of the graph indicates when the lights were on and the dark aspect indicates when the lights were off. **B** Total movement calculated for the light and dark phase (indicated by AM and PM, as well as the light and dark aspects) revealed no difference between the groups (*p = *0.25, f = 1.40, df = 24) (*n* = 7/group) and no interaction with time (*p = *0.38, f = 0.79, df = 24). As expected, there was, a significant effect of time such that animals were active during the dark phase (*p = *0.003, *f = *10.57, df = 24). A two-way ANOVA with repeated measures was used to assess statistical associations in **B**. Error bars indicate standard error of the mean

The EPM and OF constitute novel stressful environments. As such our next question was whether this hyperactivity was representative of higher habitual activity (used here to reference the animal’s constitutive home cage activity, as opposed to the activity formally measured in the OF and EPM). or specifically related to the novel environment. In-cage behavioural monitoring using the TSE PhenoMaster system tracked movement over a 24-h period (two consecutive 24-h periods were averaged for each animal). Monitoring revealed no differences in habitual activity between groups in cumulative movement (Fig. 5), movement during light/dark phases (*p* = 0.25, *f* = 1.40, df = 24) (Fig. 5B), or movement associated with grooming behaviours (t-statistic = − 0.05, *p* = 0.96 and 0.73, respectively, df = 12) (Additional file 2: Fig. 5A and B). As expected, there was, a significant effect of time (*p* = 0.003, *f = *10.57, df = 24) such that animals were more active during the dark phase (Fig. 5B). Detailed analysis of other habitual and circadian behaviour patterns showed no differences in food intake, calorie expenditure and fuel usage (Additional file 2: Fig. 5C-E). Finally, there were no differences in diurnal plasma corticosterone concentrations (main effect of MMS; F = 0.453 and p = 0.506, main effect of time (i.e. AM/PM); F = 5.512 and p = 0.025 interaction effect; F = 1.37 and *p = *0.26), total body weight (*p = *0.144), lean body mass (*p = *0.553) or fat mass (*p = *0.364).

### MMS does not associate with persistent changes in candidate gene expression or DNA methylation in the adult hypothalamus.

We next evaluated the expression of genes associated with stress signalling in the adult hypothalamus. We identified no changes in the expression of *GR* (t-statistic = 0.96, *p = *0.35, df = 15), *MR* (t-statistic = 0.31, *p = *0.76, df = 18), *Per1* (t-statistic = 0.26, *p = *0.80, df = 15) or *FKBP5* (t-statistic = 0.36, *p = *0.72, df = 15) (Additional file 2: Fig. 6A). Further, there were no changes in the expression of stress-associated genes elsewhere in the HPA axis including the adrenal and pituitary glands (Additional file 2: Fig. 6B and C). We did however find changes in expression of the *GR* (t-statistic = − 3.41, *p = *0.003, df = 18), *FKBP5* (t-statistic = − 3.31, *p = *0.003, df = 18) and *Per1* (t-statistic = − 3.29, *p = *0.004, df = 20) in the hippocampus (Additional file 2: Fig. 6D).

## Discussion

We show that ELS is associated with profound early changes in the hypothalamic DNA methylome and with changes in stress-induced behaviour in young adulthood. Animal models can be useful in furthering our understanding of the long-term effects of ELS and in facilitating the dissection of underlying mechanisms, however the large differences in experimental design and behavioural outcomes make interpretation difficult [47]; indeed, previous studies using maternal separation paradigms have described conflicting effects on behaviour [47]. We used a modified model of maternal stress [26] involving shorter periods of separation and frequent manipulation during the separation, with the lack of effects on weight gain and blood glucose levels supporting that MMS is a mild stressor and that maternal care is maintained. We speculate that the active manipulation component of MMS may lead to more consistent experience of the stress between pups and reduce heterogeneity in adult behavioural outcomes. Further, this model avoids hypoglycaemia as a confounder, which is important since hypoglycaemia is independently associated with atypical neurodevelopment [25].

Animal models of ELS have been associated with an array of behavioural phenotypes including alterations in anxiety-like behaviours [48], social interaction [49] and learning [50]. Previous studies have also hinted at stress-specific effects on behaviour following ELS. For instance, ELS in rodents has been associated with reduced memory in the object recognition task [51] but increased memory following fear conditioning [52]. In this study we used in cage behavioural monitoring to demonstrate that the hyperactivity seen in the OF and EPM following MMS was not representative of habitual increased levels of activity and as such indicates a stress-specific hyperactive phenotype. This is important as abnormal stress responses are present in a variety of neurodevelopmental and psychiatric disorders, including anxiety disorders [53], ASD [54], schizophrenia [55], bipolar disorder [56] and are also a consequence of ELS [6] and preterm birth (PTB) [12, 57, 58]. In humans, ELS has been associated with alterations in emotional regulation, reward processing, cognition and memory [59, 60]. Both ELS [61, 62] and PTB are associated with an increased incidence of ADHD [63]. Therefore, a plausible interpretation of our findings is that the ELS associated with neonatal manipulation plays a role in programming future stress response and that this is one potential mechanism underpinning the development of psychiatric disorders.

DNA methylation is dynamic during human brain development [64] and perturbations in DNA methylation are associated with several neurodevelopmental disorders, including ASD [65], schizophrenia [66] and ADHD [67]. A number of studies have reported that ELS is associated with differences in DNA methylation at loci which may affect HPA axis feedback [24, 68, 69]. We found substantial changes in hypothalamic DNA methylation immediately after MMS, and although this was not associated with widespread differences in gene expression, we did find a transcriptional signature of increased neuronal activation and an enrichment of DMRs in synapse-associated genes. In the adult brain neuronal activity is a potent modifier of DNA methylation [21], and DNA methylation has crucial roles in regulating synapse-associated genes [70]. Thus, it is possible that MMS-induced neuronal signalling may be one mechanism driving the early methylation changes. Considering the dynamic nature of DNA methylation during brain development [64] and the important role of the hypothalamus in HPA axis regulation [18], it is also possible that these early changes could be involved in programming stress-specific hyperactivity in adulthood.

Glucocorticoids are a primary mediator of the stress response, but MMS did not result in increased plasma corticosterone concentrations at P6. The classic mechanism of glucocorticoid action involves transcriptional alterations following GR binding, and an initial candidate approach to assess gene expression revealed increased expression of the glucocorticoid sensitive gene and circadian regulator, *Per1*. However, transcriptome-wide analysis revealed minimal changes in gene expression, and transcription factor binding enrichment analysis did not show enrichment for glucocorticoid binding elements. This suggests a limited role for traditional glucocorticoid-mediated transcriptional changes following MMS, in line with the well-characterised stress hyporesponsive period in neonatal rodents [71]. The lack of change in corticosterone in adulthood is perhaps not surprising given the lack of differences in the in-cage parameters and supports the suggestion that the observed behaviours are induced by exposure to a novel environment but are absent at rest.

PTB is a profound early life stressor and is associated with an increased risk of neurodevelopmental and psychiatric disorders. This is of great importance for public health policy, since PTB accounted for 10.6% of births worldwide in 2014 [72] and the rate has increased annually since 2014 in the United States [73]. One of the major long-term consequences of PTB is cognitive impairment [74] and PTB is also closely associated with autism spectrum disorder (ASD) [75], schizophrenia [76], attention deficit hyperactivity disorder (ADHD) [63] and various psychiatric disorders [77]. In mice, brain development at birth is roughly equivalent to that of a human at 24 weeks post-conception and matures to term equivalence by P10 [78], so that the time-points utilised in this study are of relevance to neurodevelopmental time-points in infants born preterm and our findings may have significance for infants born preterm. We and others have shown alterations in DNA methylation at key neurodevelopmental loci in infants born preterm in comparison to term born infants [79] although by necessity these studies have been done in peripheral tissues and whether there are also differences in relevant brain areas is unknown. Of note, preterm infants are also at increased risk for ADHD [63, 80] and although the mechanisms linking PTB with ADHD are unknown, hypotheses which have been advanced include ELS and HPA axis dysregulation [63]. Whether ADHD following PTB is particularly responsive to novel environments is unknown.

The primary limitation of this study is the use of only male animals. We chose to focus on males initially since in humans, males are more sensitive to neonatal stress associated with painful procedures [12] and animal studies suggest prenatal or early life stress may preferentially affect behavioural outcome in males [47, 81, 82]. However, in humans and animal models females have an increased risk of depression and anxiety disorders in later life following childhood stress [83, 84]. As such, future studies should focus on the investigation of outcome in both sexes following MMS.

In this study we used meDIP seq to identify DMRs associated with MMS. Although there is generally good local correlation between methylated cytosine residues [85], validation of our findings in future studies using methods with higher resolution such as whole genome bisulphite sequencing will be important. Furthermore in this study, sequencing was carried out on bulk hypothalamic tissue at a single timepoint. Future experiments should prioritise investigation of the transcriptome and DNA methylome within individual nuclei and at cellular resolution. Understanding the persistence of the DNA methylation changes we outline in the neonatal period, into adulthood will also be an important future consideration. Exploring the causal relationship of neonatal changes in DNA methylation to subsequent behaviour through emerging experimental, locus-specific modulation of DNA methylation [86] also promises to provide many exciting insights. Finally, in this study we investigated the hypothalamus, as it is a core modulator of the HPA axis and thus stress responses. However, it is clear that complex behaviours resulting from ELS are the result of contributions from several brain areas and neural circuits, including those in corticolimbic and striatal brain regions [87, 88]. Future studies should aim to evaluate these regions and to understand how they interact in the generation of complex behaviours in the context of ELS.

In conclusion, we have demonstrated differential methylation in the hypothalamus in the neonatal period and stress-induced hyperactivity in adulthood following ELS. We suggest that MMS is a useful model for the study of stress-associated alterations in brain development and may be of particular relevance to PTB.

## Supplementary Information

**Additional file 1: Table S1**. List of qPCR primers and associated UPL probe used in this manuscript. **Table S2** List of genes identified by 3’ mRNA sequencing with a logFC greater than 0.5. **Table S3** List of DMRs identified by meDIP sequencing with an FDR less than 0.05.

**Additional file 2: Fig. S1.** MDS plot and transcription factor binding enrichment analysis for 3’ mRNA sequencing. **A** MDS plot for all sequenced samples (control-blue and MMS-orange). Dimension 1 accounted for 50% of the total variance in the dataset, while dimension 2 accounted for 15%. **B** Transcription factor binding enrichment analysis using the oPOSSUM software, identifies transcription factors with binding sites overrepresented among genes which have a logFC > 1.5. Listed are the Fisher (clear bars) and Z scores (black bars) for each transcription factor. Statistical comparisons were done using an independent t-test. Error bars indicate standard error of the mean, *n* = 10/group. **Fig. S2** meDIP sequencing. **A** Venn diagram of 2 methods of analysis for differential DNA methylation shows significant overlap between DESeq2 and edgeR. There were 493 and 7047 unique DMRs associated with the edgeR and DESeq2 methods, respectively, with 12,456 DMRss identified in both methods. **B** There was no correlation between DMRs (y-axis) and expression of corresponding genes (logFC > 0.5; x-axis). Pearson correlation coefficient 0.07; *p = *0.36. **Figure S3** EPM. **A** The MMS group (black circles) moved faster than the control group (clear circles) (*p = *0.02, df = 20). **B** The MMS group spent more time mobile (*p = *0.02, df = 20), total test duration 300 s. **C** The MMS group travelled further in the closed arm (*p = *0.048, df = 20), but **D** there was no difference in the duration of each visit to the closed arm (*p = *0.06, df = 20). There were no differences in the distance travelled in the open arms of the maze (E; *p = *0.38, df = 20) or the average duration of visit to the open arms (F; *p = *0.90, df = 20). All comparisons were made using an independent t-test. Error bars indicate standard error of the mean, *n* = 11/ group. **Fig. S4** OF and tail suspension tests. **A** Animals in the MMS group (black circles) moved with a higher speed throughout the testing period when compared to controls (clear circles) (*p = *0.02, df = 20). **B** The MMS group spent more time mobile (*p = *0.02, df = 20), total test time 300 s. **C** The MMS group travelled further in the outer zone (*p = *0.04, df = 20). **D** The average visit to the outer zone was shorter in the MMS group (*p = *0.048, df = 20). **E** The MMS group also travelled further in the inner zone (*p = *0.02, df = 20). **F** There was no difference between groups with respect to duration of visit to the inner zone (*p = *0.48, df = 20). **G** In the tail suspension test, there was no difference in total immobile time between groups (*p = *0.32, df = 20). Total length of testing 360 s. All comparisons were made using independent t-tests. Error bars indicate standard error of the mean, *n* = 11/ group. **Fig. S5.** In cage analysis reveals no statistical differences in in-cage activities, feeding behaviour and calorie expenditure following MMS. There were no differences in (**A**) x-axis or (**B**) y-axis related grooming behaviour as quantified by laser beam breaks in the specified plane (*p = *0.96 and 0.73, respectively, df = 12). **C** There was no difference in food intake quantified as total feeding during lights on (control AM and MMS AM) and lights off (control PM and MMS PM). There was a significant effect of time (*p* > 0.001), but no effect of MMS (*p = *0.75) or interaction between time and MMS (*p = *0.15). **E** There was no difference in calorie expenditure normalised for lean body mass (*p = *0.99, df = 12) or in fuel usage as indicated by respiratory exchange ratio (E; control (black line) and MMS (grey line)) over 24 h (error bars indicate standard error of the mean). N = 7/ group. **Fig. S6** No changes in candidate gene expression in the hypothalamus, adrenal or pituitary glands but there are changes in gene expression in the hippocampus at 4 months of age, following MMS. **A** There were no differences in expression of the GR (*p = *0.35, df = 15), MR (*p = *0.76, df = 18), Per1 (*p = *0.80, df = 15) or FKBP5 (*p = *0.72, df = 15) in the hypothalamus between control (clear circles) and MMS (black filled circles) mice at 4 months of age. **B** In the adrenal gland, there were no changes in gene expression for Cyp11b1 (*p = *0.35, df = 15), MC2R (*p = *0.72, df = 17) or stAr (*p = *0.80, df = 16) between the control (clear circles) and MMS (black filled circles) groups. **C** There were no changes in candidate gene expression in the pituitary gland for the GR (*p = *0.81, df = 20), the MR (*p = *0.13, df = 19), FKBP5 (*p = *0.92, df = 14), Per1 (*p = *0.09, df = 20) or POMC (*p = *0.78, df = 18) between control (clear circles) and MMS (black filled circles) groups. **D** In the hippocampus, there was increased expression of GR (*p = *0.003, df = 18), FKBP5 (*p = *0.003, df = 18) and Per1 (*p = *0.004, df = 20), but no change in expression of the MR (*p = *0.37, df = 19) or HSD11b1 (*p = *0.08, df = 20). All candidate gene expression was normalised to the expression of TBP. All statistical comparisons were made using independent t-tests; n = 7–11/group. Error bars indicate standard error of the mean.

## Data Availability

The datasets supporting the conclusions of this article are available in the GEO repository under accession numbers GSE147375 and GSE146892.
