# Supplementary material for: Altered hypothalamic DNA methylation and stress-induced hyperactivity following early life stress
Source: Epigenetics Chromatin. 2021 Jun 30;14:31. doi: 10.1186/s13072-021-00405-8 (PMC8247254; doi:10.1186/s13072-021-00405-8)
Supplement: Supplementary file 2 — Additional file 2: Fig. S1. MDS plot and transcription factor binding enrichment analysis for 3’ mRNA sequencing. A MDS plot for all sequenced samples (control-blue and MMS-orange). Dimension 1 accounted for 50% of the total variance in the dataset, while dimension 2 accounted for 15%. B Transcription factor binding enrichment analysis using the oPOSSUM software, identifies transcription factors with binding sites overrepresented among genes which have a logFC > 1.5. Listed are the Fisher (clear bars) and Z scores (black bars) for each transcription factor. Statistical comparisons were done using an independent t-test. Error bars indicate standard error of the mean, n = 10/group. Fig. S2 meDIP sequencing. A Venn diagram of 2 methods of analysis for differential DNA methylation shows significant overlap between DESeq2 and edgeR. There were 493 and 7047 unique DMRs associated with the edgeR and DESeq2 methods, respectively, with 12,456 DMRss identified in both methods. B There was no correlation between DMRs (y-axis) and expression of corresponding genes (logFC > 0.5; x-axis). Pearson correlation coefficient 0.07; p = 0.36. Figure S3 EPM. A The MMS group (black circles) moved faster than the control group (clear circles) (p = 0.02, df = 20). B The MMS group spent more time mobile (p = 0.02, df = 20), total test duration 300 s. C The MMS group travelled further in the closed arm (p = 0.048, df = 20), but D there was no difference in the duration of each visit to the closed arm (p = 0.06, df = 20). There were no differences in the distance travelled in the open arms of the maze (E; p = 0.38, df = 20) or the average duration of visit to the open arms (F; p = 0.90, df = 20). All comparisons were made using an independent t-test. Error bars indicate standard error of the mean, n = 11/ group. Fig. S4 OF and tail suspension tests. A Animals in the MMS group (black circles) moved with a higher speed throughout the testing period when compared to controls (clear circles) (p = [file 13072_2021_405_MOESM2_ESM.pptx]

## Slide 1
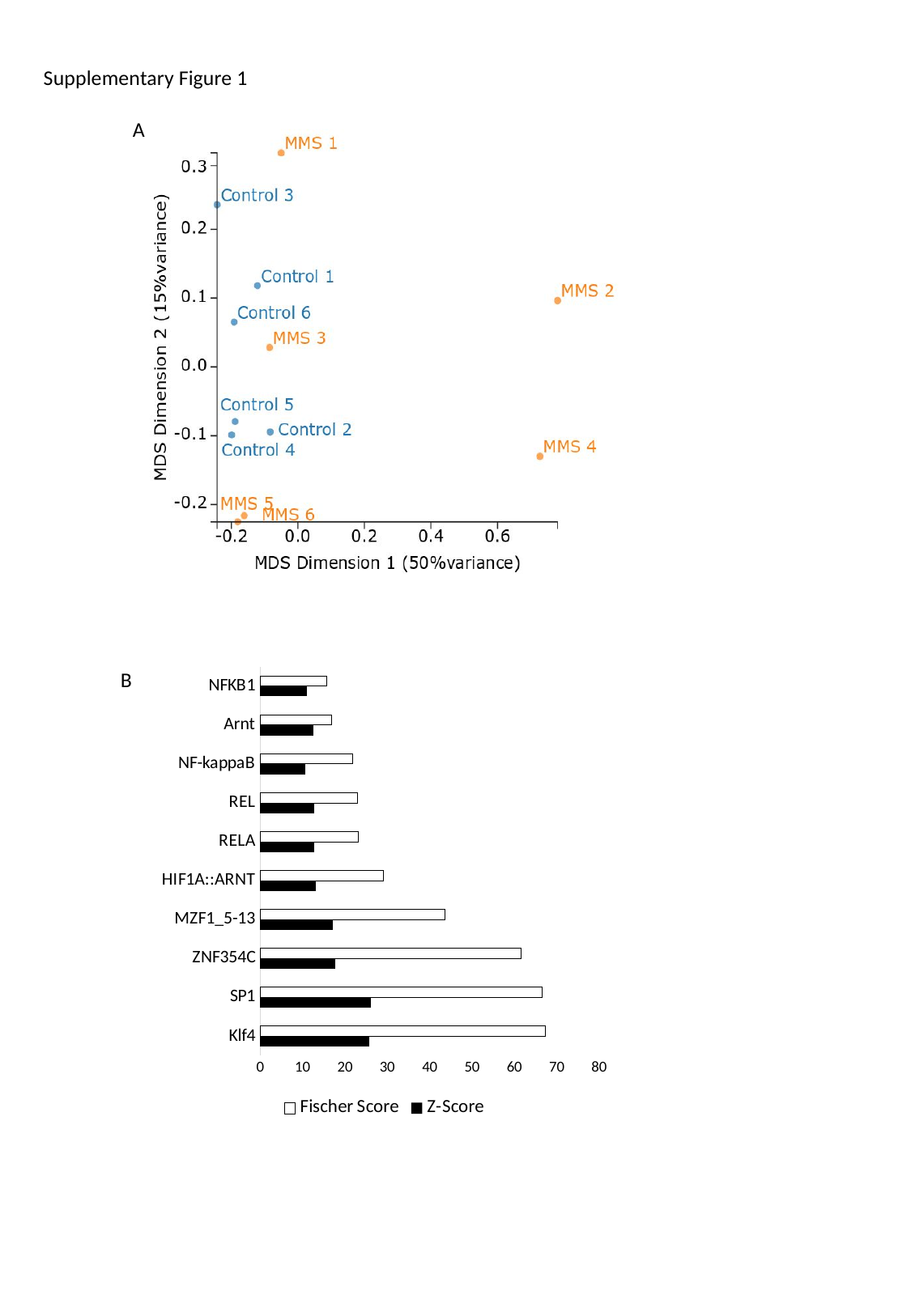

Supplementary Figure 1
A
### Chart
| Category | Z-Score | Fischer Score |
|---|---|---|
| Klf4 | 25.768 | 67.297 |
| SP1 | 26.101 | 66.646 |
| ZNF354C | 17.784 | 61.64700000000001 |
| MZF1_5-13 | 17.113 | 43.58600000000001 |
| HIF1A::ARNT | 13.145 | 29.029 |
| RELA | 12.761 | 23.255 |
| REL | 12.68 | 22.999 |
| NF-kappaB | 10.671 | 21.77199999999999 |
| Arnt | 12.64 | 16.786 |
| NFKB1 | 11.101 | 15.622 |B

## Slide 2
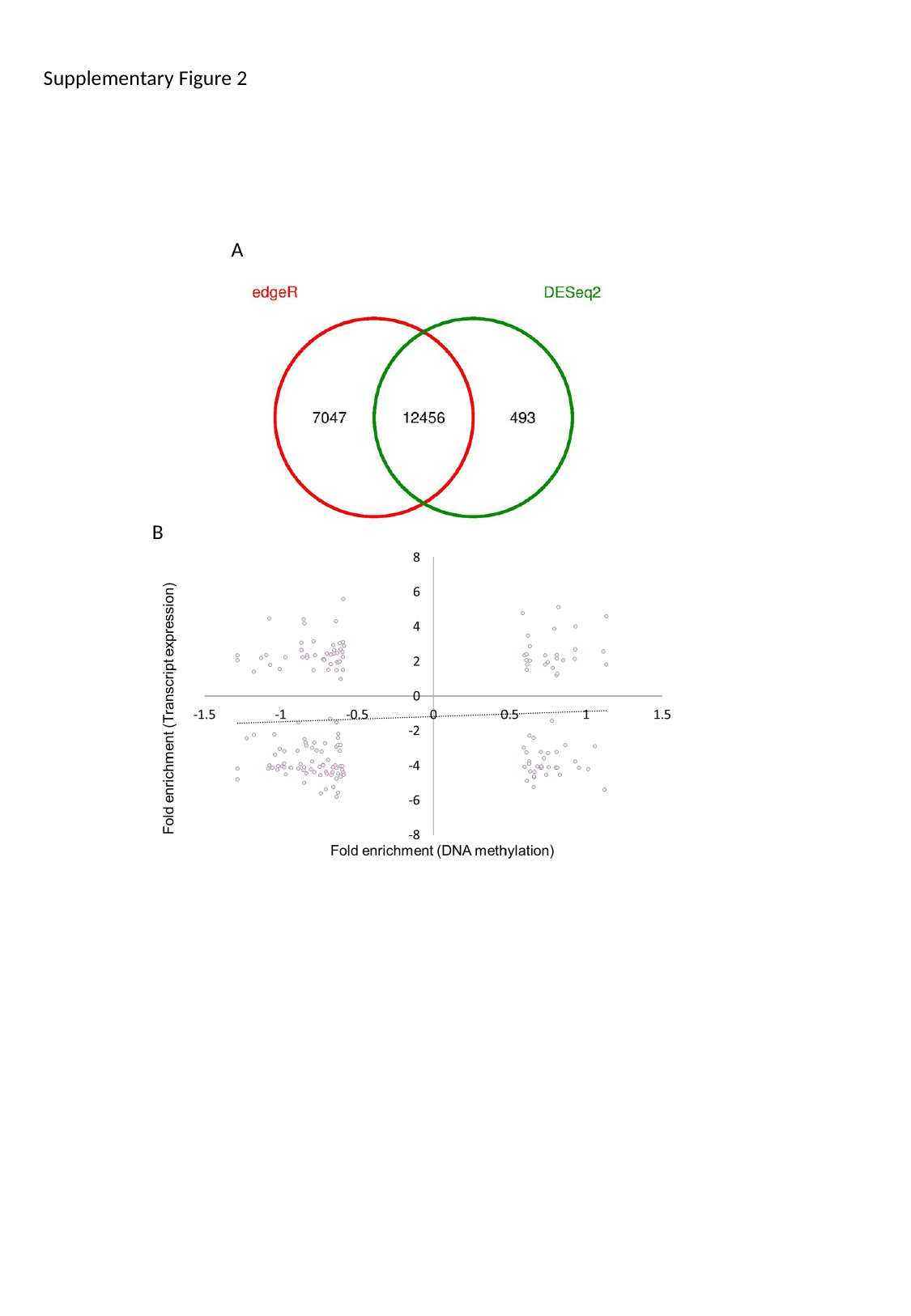

Supplementary Figure 2
A
B

## Slide 3
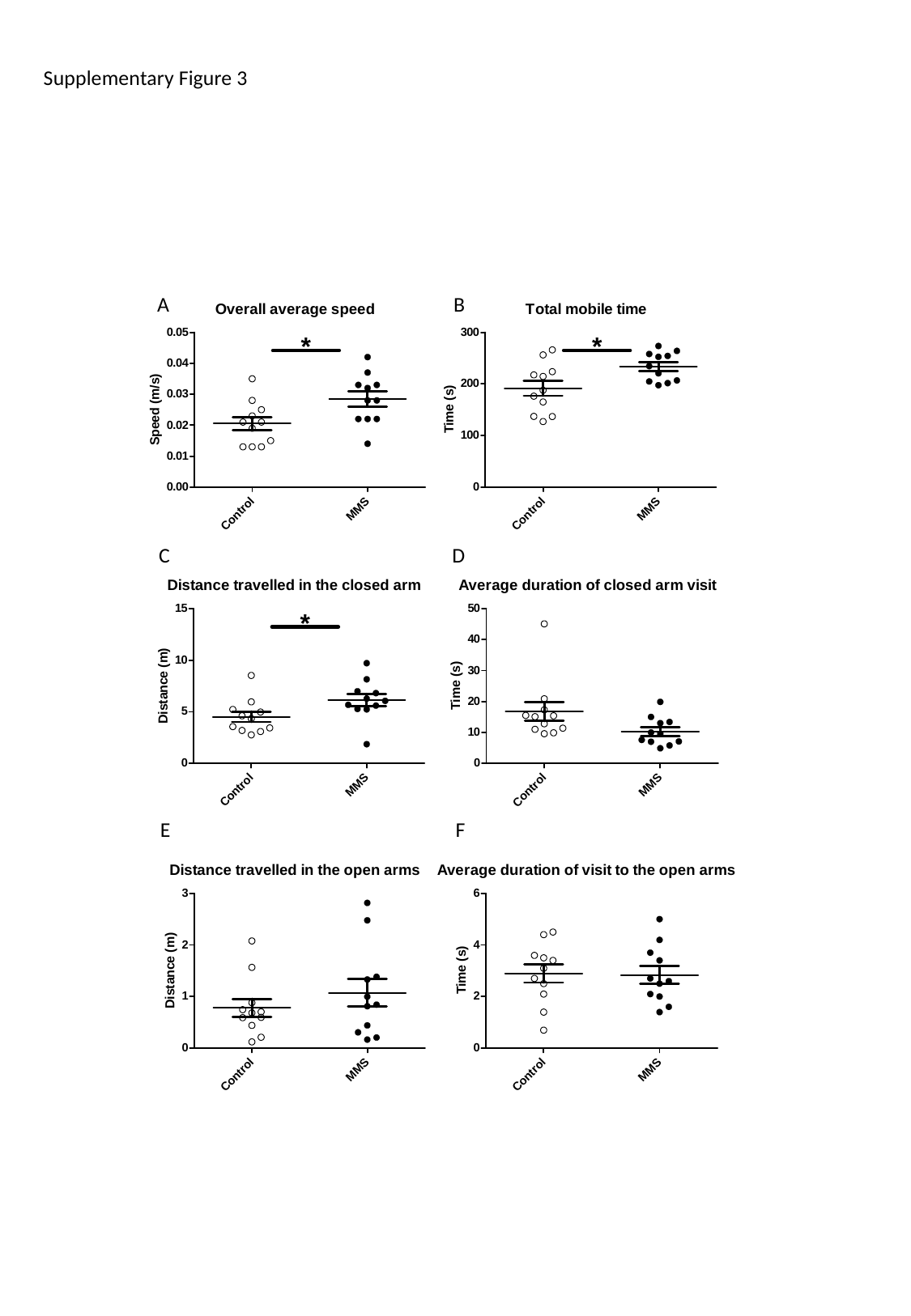

Supplementary Figure 3
B
A
D
C
E
F

## Slide 4
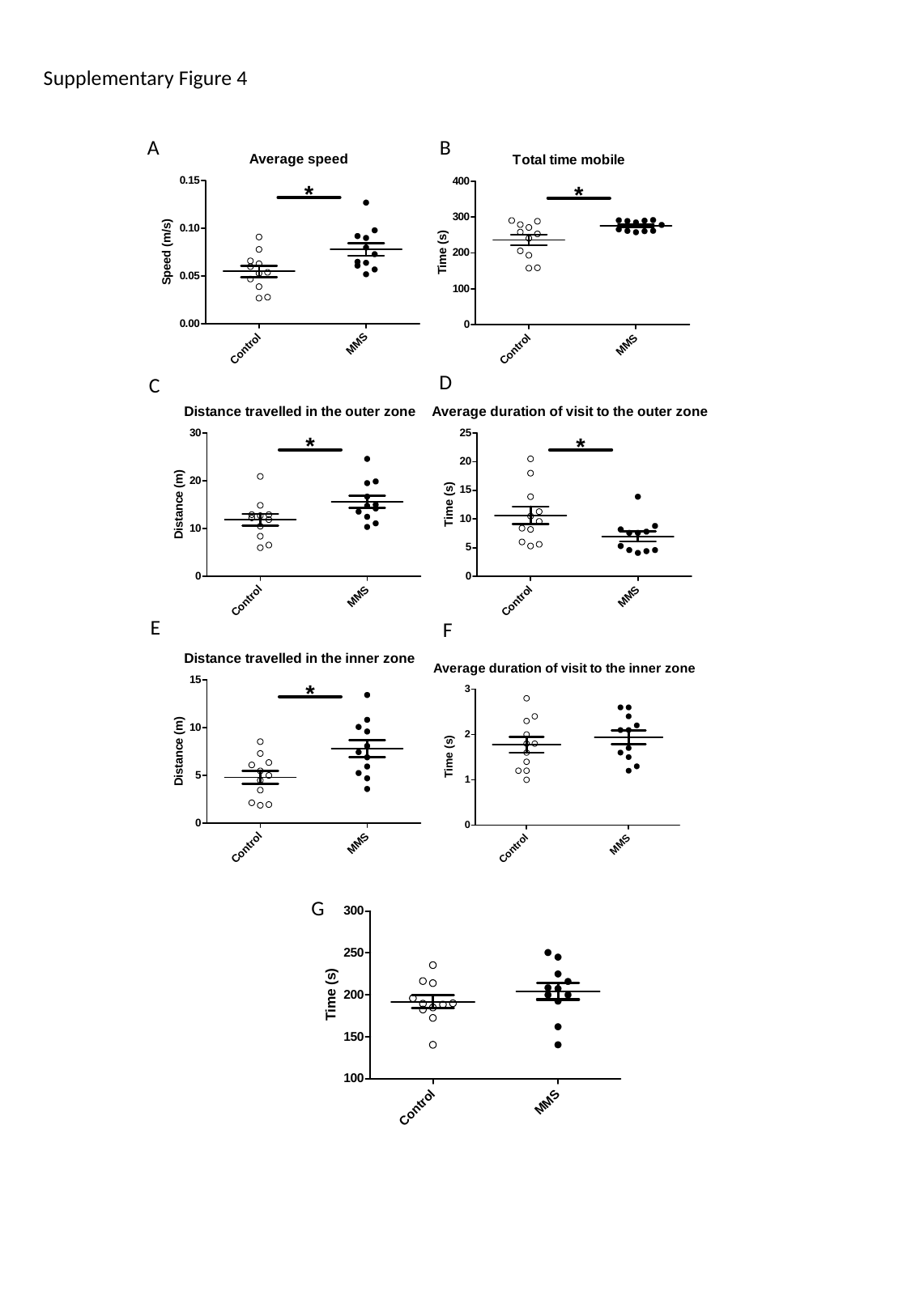

Supplementary Figure 4
B
A
D
C
E
F
G

## Slide 5
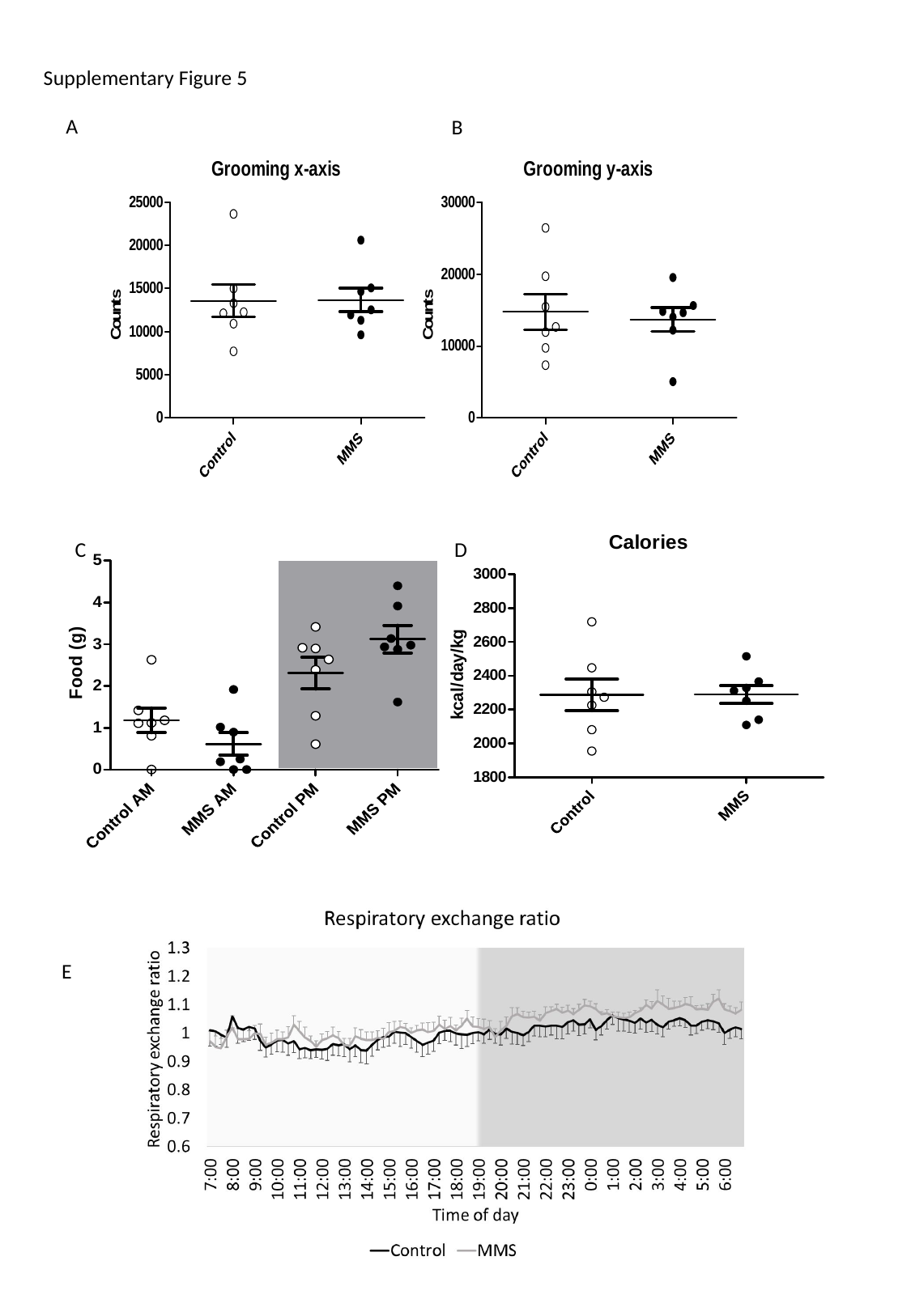

Supplementary Figure 5
A
B
C
D
E

## Slide 6
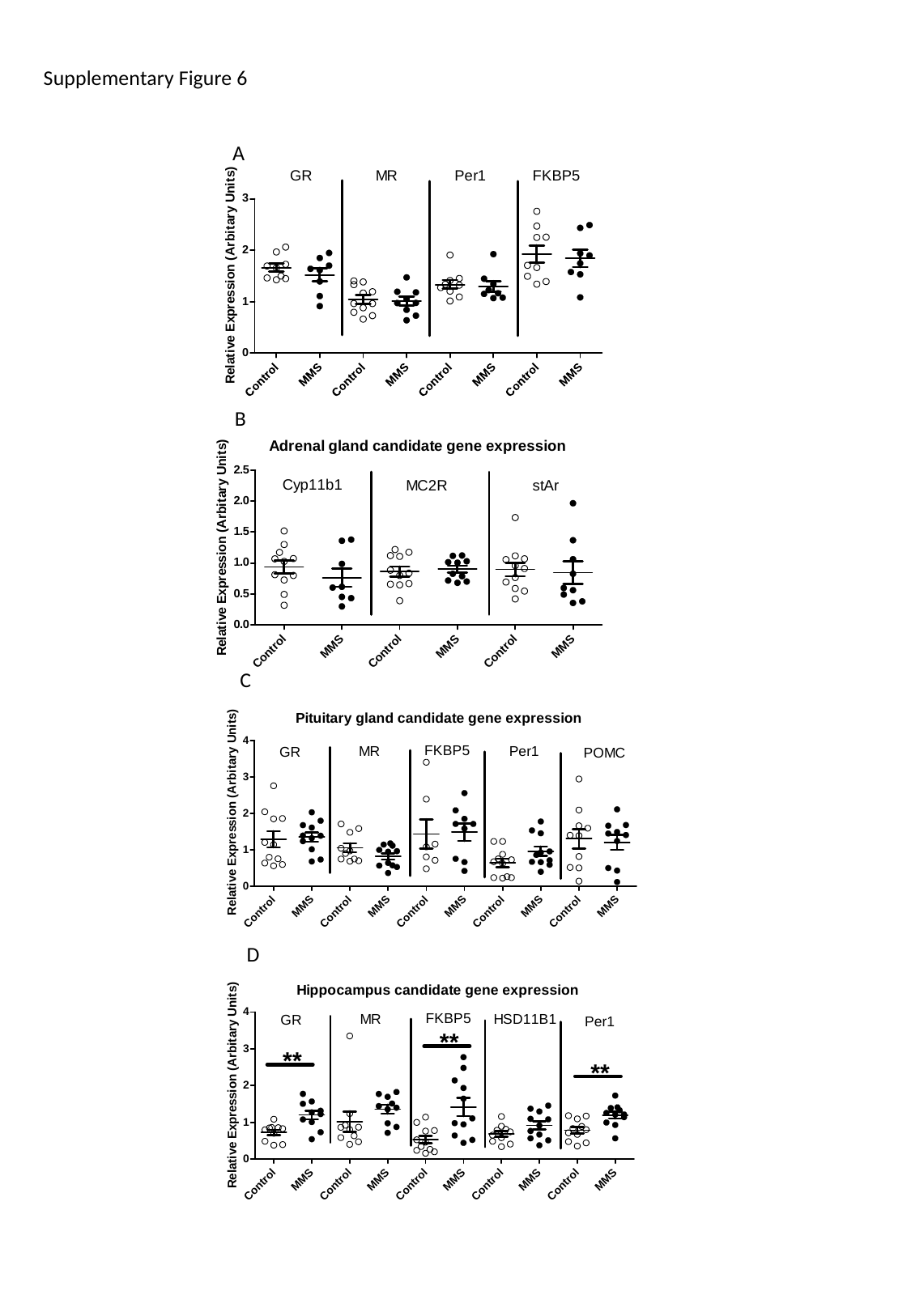

Supplementary Figure 6
A
B
C
D
